# Joint Time-of-Arrival and Carrier-Phase Measurement and Tracking for Enhanced Loran Signals in Complex Interference Environments

**DOI:** 10.3390/s26123623

**Published:** 2026-06-06

**Authors:** Ziming Yuan, Shuaihe Gao, Pengfei Li, Shougang Zhang

**Affiliations:** 1 National Time Service Center, Chinese Academy of Sciences, Xi’an 710600, China; yuanziming24@mails.ucas.ac.cn (Z.Y.); zhangshougang@ntsc.ac.cn (S.Z.); 2 University of Chinese Academy of Sciences, Beijing 100039, China; 3 School of Measurement-Control Technology and Communications Engineering, Harbin University of Science and Technology, Harbin 150080, China; lpf5356@hrbust.edu.cn; 4 Hefei National Laboratory, Hefei 230088, China

**Keywords:** eLoran, TOA estimation, carrier-phase tracking, Kalman filtering, multiple-model adaptive filtering, outlier suppression

## Abstract

**Highlights:**

**What are the main findings?**

- A joint time-of-arrival (TOA) and carrier-phase tracking method is developed for enhanced Loran (eLoran) receivers operating under low signal-to-noise ratio (SNR) and dynamic conditions.
- The proposed method improves TOA convergence, preserves phase continuity, and provides more stable frequency-offset estimation than a conventional Costas-loop-based carrier tracking method.

**What are the implications of the main findings?**

- The method enhances the robustness and long-term stability of eLoran timing in complex interference environments.
- It offers a practical framework for high-precision terrestrial timing and synchronization applications.

**Abstract:**

To address carrier-phase loss of lock and long-term drift in frequency-offset estimation that may arise from time-of-arrival (TOA) measurements in enhanced Loran (eLoran) timing receivers under low signal-to-noise ratio (SNR) and moderate-to-high dynamics, this paper proposes a joint TOA and carrier-phase measurement and tracking method. First, transmitter identification and group repetition interval (GRI) lock are achieved by exploiting the periodic repetition of pulse groups, and epoch folding is applied to enhance effective SNR. Then, a sub-sample TOA observation is constructed via a three-stage progressive refinement procedure: energy-matching coarse estimation, coherent cross-correlation, and parabolic peak interpolation. In parallel, baseband phase observations are obtained through coherent downconversion and accumulation. A unified state-space model incorporating TOA bias, TOA drift rate, baseband phase, and frequency offset is further established to enable joint Kalman filtering of TOA and phase. Moreover, an innovation-likelihood-weighted parallel multiple-model filter combined with measurement-noise covariance inflation is introduced to suppress outlier observations. Simulations show that the TOA estimate converges within about 1 s while maintaining phase continuity and stable frequency-offset estimation, and that the proposed method achieves superior overall robustness and long-term stability compared with a conventional Costas loop.

## 1. Introduction

Global Navigation Satellite Systems (GNSSs) have become the core infrastructure for modern Positioning, Navigation, and Timing (PNT) services. However, GNSS signals are inherently low-power and thus highly susceptible to interference and spoofing, leading to pronounced vulnerabilities in complex interference environments and security-sensitive scenarios. To enhance the security, continuity, and reliability of timing and navigation services in national defense and civil applications, building terrestrial backup systems that can operate independently of satellite navigation systems has become an international consensus. Enhanced Loran (enhanced Loran, eLoran)—also referred to as Enhanced Long-Range Navigation—operates in the 90–110 kHz low-frequency (LF, longwave) band and relies on high-power groundwave propagation [1]. With wide-area coverage, strong interference resilience, and an infrastructure independent of GNSS, eLoran is widely recognized as an important terrestrial alternative and backup for GNSS timing and navigation services.

The eLoran employs a pulsed modulation scheme with a carrier center frequency of approximately 100 kHz, and the signal propagates predominantly as a groundwave. Affected by factors such as ground electrical properties, variations in propagation paths, and receiver local-oscillator (LO) stability, the eLoran carrier phase cannot be stably mapped one-to-one to geometric range [2]; therefore, it is not well suited to serve as an independent high-precision ranging observable. In general, carrier-phase observations have a higher effective signal-to-noise ratio than time-of-arrival (TOA) measurements. In addition, compared with TOA measurements, carrier-phase observations exhibit better continuity over short time scales. As a result, carrier phase can be used to provide auxiliary constraints on TOA measurements, improving timing stability and receiver performance under weak-signal conditions. Consequently, achieving robust and continuous carrier-phase tracking under low-SNR and time-varying dynamics is a key issue in the design of high-performance eLoran digital receivers.

For carrier phase, “dynamics” essentially refer to how rapidly the phase evolves over time. It is commonly characterized by the first-order time derivative (phase rate) or the second-order derivative (phase acceleration). When either the phase rate or phase acceleration varies rapidly, the scenario is considered a high-dynamics condition. In eLoran systems, sampling and synchronization jitter caused by receiver oscillator instability, as well as perturbations in the propagation path, can likewise be equivalently regarded as high-dynamics effects.

For a conventional Costas loop, the loop bandwidth $B_{L}$ directly determines the trade-off between dynamic tracking capability and noise suppression [3]. On the one hand, a larger loop bandwidth improves the loop response to frequency offsets and rapid phase variations, thereby reducing the dynamic stress error. On the other hand, a larger bandwidth allows more in-band noise to enter the loop, resulting in increased phase jitter. Conversely, a smaller loop bandwidth helps suppress phase noise, but reduces the loop’s ability to track frequency offsets and dynamic variations. Under moderate-to-high dynamic conditions, this may lead to residual frequency bias and long-term phase drift.

Theoretically, the phase tracking error variance of a Costas loop is generally related to the loop bandwidth and the carrier-to-noise density ratio, and can be approximately expressed as [4]

(1) $$\sigma_{\phi }^{2}\propto \frac{B_{L}}{C/N_{0}}$$

where $B_{L}$ is the loop noise bandwidth and $C/N_{0}$ is the carrier-to-noise density ratio. This relationship indicates that, for a given signal-to-noise condition, increasing the loop bandwidth increases the output phase noise. Under low-SNR conditions, even with the same loop bandwidth, the phase jitter increases significantly. Meanwhile, the dynamic stress error generally increases as the loop bandwidth decreases. Therefore, a single fixed-bandwidth Costas loop is difficult to satisfy both low-noise tracking and robust dynamic tracking requirements [2]. As an optimal recursive state-estimation method, the Kalman filter can model carrier phase and its dynamics within a unified state-space framework and can be adaptively tuned via the process-noise and measurement-noise covariance matrices [5]; in recent years, it has demonstrated strong performance in carrier tracking for communications and GNSS applications.

However, in many practical applications, insufficient modeling of certain noise components may cause the Kalman filter’s error covariance matrix to lose symmetry and positive definiteness after repeated updates, potentially leading to filter divergence [6]. Therefore, optimal Kalman filter operation requires that the system state variables be sufficiently observable and that process-noise modeling be reasonably accurate, so that the filter parameters can be tuned toward their optimal values.

To address the above issues, this paper investigates carrier-phase-aided TOA tracking for eLoran timing receivers. A Kalman-filter-based joint estimation method is proposed, and correlation-based processing is used to perform GRI search and accurate TOA estimation. On this basis, to overcome the limited robustness of a single Kalman filter model under complex noise and time-varying system parameters, a parallel multiple-model filtering framework and an inflation-threshold (covariance-inflation with gating) mechanism are further introduced to enhance adaptability to different signal dynamics and noise environments [7]. Through simulations of realistic eLoran signal reception, comparative results demonstrate that the proposed method outperforms the conventional Costas loop in carrier-phase continuity, frequency-offset estimation accuracy, and timing stability. The proposed framework provides a robust and extensible implementation approach for carrier-phase-aided timing in eLoran systems and offers a useful reference for high-precision synchronization of terrestrial timing systems in complex electromagnetic environments.

## 2. Correlation Between TOA and Carrier Phase in eLoran Signals

The eLoran single-pulse signal follows the standard Loran pulse shape (often referred to as the Chayka pulse) with a 100 kHz carrier, and it is a phase-modulated pulse. If a single pulse is modeled as an envelope-modulated carrier, it can be written as [8]:

(2) $$x\left(t\right)=\;e_{g}\left(t-\tau_{k}\;\right)\cdot \sin\left(2\pi f_{c}\;\left(t-\tau_{k}\right)+\phi_{k}\right)$$

where the envelope pulse $e_{g}\left(t-\tau_{k}\right)$ is a smooth function formed by a quadratic rise followed by an exponential decay; $\tau_{k}$ denotes the propagation delay of the k-th epoch relative to the receiver time reference, and $\phi_{k}$ is the transmitted phase of that pulse. In the eLoran scheme, $\phi_{k}$ is typically determined by phase coding (taking values 0 or π) to convey data.

For eLoran signals, the TOA measurement and the baseband carrier-phase observation essentially reflect the same propagation delay $\tau_{k}$, but they represent it in different ways with different precision and effective bandwidth. For eLoran signals with a carrier frequency of approximately 100 kHz, one carrier cycle corresponds to about 10 μs in time delay. Therefore, carrier-phase observations are sensitive to delay variations well below one carrier cycle, typically at the sub-microsecond to microsecond level. In general, the TOA measurement ${\hat{\tau }}_{k}$ and the carrier-phase measurement ${\hat{\Phi }}_{k}$ can be expressed as [9]:

(3) $${\hat{\tau }}_{k}=\;\tau_{k}\;+\;v_{\tau ,k}$$

(4) $${\hat{\Phi }}_{k}=-2\pi f_{c}\tau_{k}+\phi_{0}\left(k\right)+v_{\Phi ,k}$$

where $v_{\tau ,k}$ and $v_{\Phi ,k}$ denote the TOA measurement noise and the phase measurement noise, respectively; $-2\pi f_{c}\tau_{k}$ is the carrier-phase term introduced by the propagation delay; and $\phi_{0}\left(k\right)$ is an equivalent baseband phase term independent of the propagation delay, which aggregates contributions from the transmitted phase coding, the receiver local-oscillator (LO) initial phase and its phase noise, the integrated frequency-offset term, and other effects [10].

Equation (4) further describes the carrier-phase observation corresponding to the same propagation delay $\tau_{k}$ in Equation (3), where the delay term is mapped into the carrier-phase domain through $-2\pi f_{c}\tau_{k}$ [11]. As implied by (3) and (4), directly observing $\tau_{k}$ via TOA has the advantages of being absolute and free from cycle ambiguity (including π ambiguity). However, the accuracy of TOA measurements is limited by the effective signal bandwidth, sampling rate, and the resolution of correlation-peak localization. Consequently, compared with carrier-phase observations, TOA measurements usually exhibit larger measurement noise, especially over short time scales. By contrast, the carrier phase is extremely sensitive to small changes in propagation delay, offering higher resolution and good short-term continuity. However, the carrier-phase observation is not an absolute delay measurement. In eLoran, the transmitted pulses commonly employ BPSK-type phase coding, in which the carrier phase may be shifted by either 0 or π according to the phase code. In addition, the measured phase is usually wrapped into a principal interval, such as (−π, π]. Therefore, without proper phase-code correction and phase unwrapping, the practical carrier-phase observation may contain π-ambiguity and apparent discontinuities [12].

**Figure 1:**
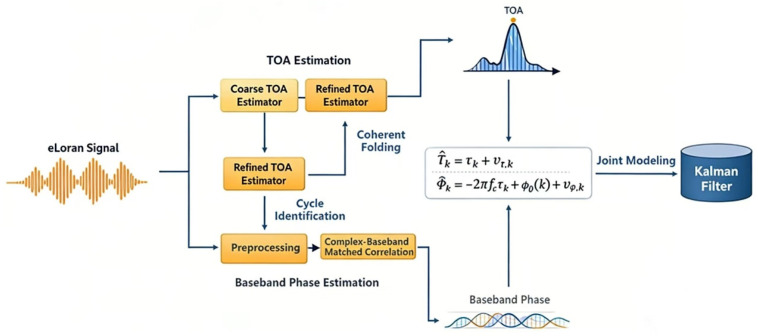
Block Diagram of the Proposed Joint TOA and Carrier-Phase Tracking Method.

Therefore, by exploiting the complementary properties of these two observables, a joint estimation framework (as illustrated in Figure 1) can be established: the carrier phase is used to constrain the smooth evolution and drift of $\tau_{k}$ [13]; meanwhile, the TOA provides an absolute delay reference to assist phase ambiguity resolution and to suppress long-term drift caused by ambiguity and accumulated frequency-offset errors, thereby enabling robust joint tracking under complex interference conditions [10].

## 3. GRI Search and TOA Estimation

The Group Repetition Interval (GRI) of an eLoran pulsed signal is defined as the time interval between two successive transmitted pulse groups from a given transmitter station. In an eLoran system, all stations within the same chain transmit signals with the same GRI; therefore, the GRI serves as a key identifier for distinguishing different chains. The GRI is specified with a resolution of 10 μs and is commonly represented by a four-digit number (with a trailing zero implicitly assumed), ranging from 4000 to 9999 [14]; every two GRIs form a Phase Code Interval (PCI). For example, the Korean eLoran system uses a GRI of 99,300 μs, whereas the North American Great Lakes chain uses a GRI of 89,700 μs.

To ensure that the receiver locks onto a unique chain and rapidly determines the GRI, the received signal is sampled at a sampling interval $T_{s}$ to obtain:

(5) $$r\left[n\right]\;=\;x\left(nT_{s}\right),\;\;\;n\in Z,n<M$$

where $T_{s}$ is the sampling interval. The term $x\left(nT_{s}\right)$ represents the sample of the continuous-time received eLoran signal $x\left(t\right)$, given in Equation (1), at the n-th sampling instant. For subsequent discrete-time signal processing, the sampled received signal is uniformly denoted as $r\left[n\right]$, and M is the total length, i.e., the number of samples, of the discrete sequence $r\left[n\right]$.

Construct a candidate GRI set $G=\left\{T_{G}^{\left(i\right)}\right\}$. For each candidate $T_{G}^{\left(i\right)}$, the corresponding discrete delay in samples satisfies $N_{i}\;=\;\lfloor T_{G}^{\left(i\right)}/\;T_{s}\rceil \;$, if $T_{G}^{\left(i\right)}$ matches the true GRI $T_{G}$, then adjacent pulse groups should be separated by $N_{i}$ samples in the sampling domain.

Based on the delay autocorrelation property, define the search metric $R\left(T_{G}^{\left(i\right)}\right)$ and scan over the candidate set [15]:

(6) $$R\left(T_{G}^{\left(i\right)}\right)\;=\;\left|\sum_{n=0}^{M-N_{i}-1}r\left[n\right]\;\cdot \;r^{*}\left[n-N_{i}\right]\right|$$

If $T_{G}^{\left(i\right)}$ is close to the true GRI, the pulse groups align and add coherently, yielding a large correlation value. A coarse estimate of the true GRI is then obtained by:

(7) $${\hat{T}}_{G}\;=\;\underset{T_{G}^{\left(i\right)}\in G}{\arg\;\max\;}R\left(T_{G}^{\left(i\right)}\right)$$

Since GRIs are standardized, if the coarse estimate ${\hat{T}}_{G}$ falls within a prescribed threshold, chain identification can be completed and the receiver adopts the standard GRI value, ${\hat{T}}_{G}=T_{G}$ is fixed thereafter.

This approach exploits the periodic repetition structure of eLoran signals and does not rely on an accurate prior pulse start time; however, it cannot distinguish different stations that share the same GRI. In addition, slow-varying uncertainties caused by the time base and propagation—such as GRI drift and sampling-clock errors—are equivalently absorbed into $\tau_{k}$ and its drift ${\dot{\tau }}_{k}$ (reflecting long-term stability) for tracking.

Therefore, in a practical system the true GRI is not strictly constant and can be written as [8]

(8) $$T_{G}={\hat{T}}_{G}+\tau_{k}$$

It should be noted that the nominal GRI specified by the eLoran chain is fixed. The discussion here does not imply that the GRI of the transmitting station itself changes. Rather, under the receiver sampling time base, the effective GRI observed in the receiver sampling domain, denoted as ${\hat{T}}_{G}$, may exhibit a small apparent deviation from the nominal GRI.

**Figure 2:**
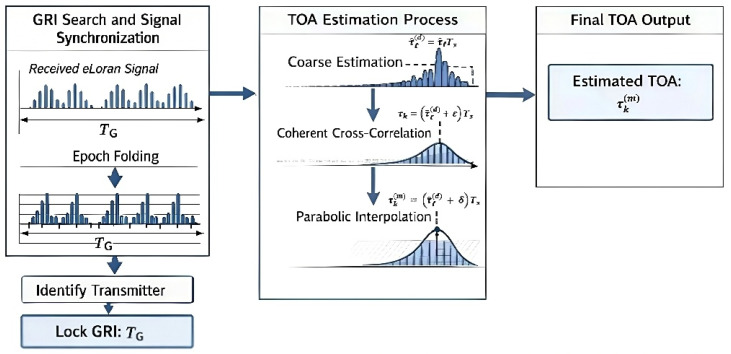
Flowchart of GRI Establishment and TOA Estimation.

GRI locking provides an accurate periodic reference for pulse-group alignment, thereby enabling coherent epoch folding, improving the effective signal-to-noise ratio, and further allowing stable TOA extraction via correlation-based processing, as illustrated in Figure 2.

At the same time, in practical eLoran systems, multiple stations may exist within the same chain, each with slightly different TOAs at the receiver. Under such circumstances, the autocorrelation-based GRI search may produce false correlation peaks, where signals from different stations superpose and generate local peaks at certain sampling points, potentially interfering with chain identification or coarse TOA estimation [16]. To mitigate this effect, the present work constrains peak selection within a plausible time window based on the known minimum TOA interval between stations, and only the global maximum peak within this window is selected from the autocorrelation spectrum to avoid local false peaks. Furthermore, by incorporating the joint TOA–carrier-phase Kalman filtering, phase continuity and smoothing constraints are applied to suppress TOA deviations caused by false correlation peaks, thereby ensuring stable subsequent tracking.

The time window can be defined as:

(9) $$\tau_{\min}\leq \tau_{k}\leq \tau_{\max},\tau_{\min}=\tau_{k-1}\;+\;\Delta_{\min},\tau_{\max}=\tau_{k-1}+\Delta_{\max}$$

where $\tau_{min}$ and $\tau_{max}$ denote the minimum and maximum allowable TOAs for the current pulse, respectively. They are determined by the TOA of the previous pulse, $\tau_{k-1}$, and the known minimum and maximum TOA intervals between stations, $\Delta_{\min}$ and $\Delta_{\max}$. Candidate peaks in the autocorrelation spectrum, $\tau_{k}$, must satisfy the constraint given in Equation (7).

Within this time window, the global maximum peak is selected:

(10) $${\hat{\tau }}_{k}^{coarse}=\underset{\tau_{k}\in \left[\tau_{\min},\tau_{\max}\right]}{\arg\;\max}R\left(\tau_{k}\right)\;$$

where ${\hat{\tau }}_{k}^{coarse}$ denotes the global maximum of the autocorrelation function $R\left(\tau \right)$ among all candidate peaks within the time window, and it is used as the coarse TOA estimate for subsequent processing.

To estimate the delay $\tau_{k}$ that includes small slow-varying biases, after locking onto the true $T_{G}$, the received signal is epoch-folded with period $T_{G}$. Let $N_{G}=\lfloor T_{G}\;f_{s}\rceil$, then the sampled signal within the k-th epoch is

(11) $$r_{k}\left[n\right]=r\left[n+kN_{G}\right],\;\;\;m\in Z,m<N_{G}-1$$

where k is an index indicating the offset from the k-th epoch to the n-th sample; $r_{k}\left[n\right]$ denotes the samples taken within the k-th epoch and may contain noise. The folding window $N_{G}$ only needs to cover the entire pulse group, typically on the order of tens to hundreds of microseconds.

The epoch-folded signal is defined as [14]

(12) $$\bar{r}\left[n\right]=\frac{1}{K}\sum_{k=0}^{K-1}r_{k}\left[n\right]$$

When the noise is approximately independent and identically distributed, the folded signal energy accumulates approximately linearly with the number of epochs, while the noise variance decreases by about $\frac{1}{K}$, which effectively improves the SNR.

Next, generate a local eLoran template signal $s\left[m\right]$ of length $N_{L}$, and compute the cross-correlation between the two signals [17]:

(13) $$C\left[d_{l}\right]\;=\;\sum_{n\;=\;0}^{N_{L}\;-\;1}\bar{r}\left[n\right]\;\cdot \;s^{*}\left[n-d_{l}\right]$$

where $d_{l}$ is the template delay. Under additive white Gaussian noise (AWGN), the cross-correlation is equivalent to matched filtering and serves as the optimal linear detector. Therefore, the correlation-peak location is:

(14) $${\hat{d}}_{l}=\;\underset{d_{l}}{\arg\;\max\;}C\left[d_{l}\right]$$

The corresponding discrete-sample-level coarse TOA estimate is

(15) $${\hat{d}}_{l}^{\left(d\right)}={\hat{d}}_{l}T_{s}$$

Were the superscript $\left(d\right)$ denotes the discrete-sample-level estimate, and ${\hat{d}}_{l}$ is the estimated discrete delay index in samples.

Since the true TOA $\tau_{k}$ is almost never exactly on an integer sample, it can be expressed as

(16) $$\tau_{k}=\left({\hat{d}}_{l}^{\left(d\right)}+\varepsilon \right)T_{s},\;\;\;|\varepsilon |<0.5$$

where ε is the sub-sample (fractional) offset relative to the nearest integer sample. Because the peak position of the cross-correlation corresponds to the signal delay and its peak shape matches the template autocorrelation, the correlation peak can be approximated by the template autocorrelation. Using the property that near the peak, the first derivative is zero and the second derivative is negative, the peak neighborhood can be locally approximated by a quadratic function:

(17) $$C\left[d_{l}\right]\;\approx \;a{\left(d_{l}\;-\;\tau_{k}\right)}^{2}\;+\;b$$

Using the peak sample and its left or right neighboring samples $C\left[{\hat{d}}_{l}\;-\;1\right]$, $C\left[{\hat{d}}_{l}\right]$, and $C\left[{\hat{d}}_{l}\;+\;1\right]$, the fractional peak offset is obtained as [18]

(18) $$\varepsilon =\frac{1}{2}\frac{C\left[{\hat{d}}_{l}-1\right]-C\left[{\hat{d}}_{l}+1\right]}{C\left[{\hat{d}}_{l}-1\right]-2C\left[{\hat{d}}_{l}\right]+C\left[{\hat{d}}_{l}\;+1\right]}$$

Under the Gaussian-noise assumption, minimizing the squared error is equivalent to maximizing the likelihood function; hence, the least-squares estimate here is a special case of the maximum-likelihood estimate, and the resulting sub-sample TOA estimate can be regarded as a maximum-likelihood estimate [19]. Let the sub-sample offset of the correlation peak relative to the integer sample be δ; the final TOA measurement can be expressed as

(19) $$\tau_{k}^{\left(m\right)}=\left({\hat{d}}_{l}^{\left(d\right)}+\delta \right)T_{s}$$

where δ denotes an estimation error smaller than v which has been constrained within half a sampling interval; therefore, δ satisfies δ ∈ [−0.5, 0.5].

## 4. Joint TOA and Carrier-Phase Tracking Model

### 4.1. Baseband Carrier Phase in Phase Observations

From the complex cross-correlation output in (13), the phase observation can be obtained from the argument of the complex correlation value at the estimated discrete delay index.

(20) $${\hat{\Phi }}_{k}=\arg\left\{\sum_{n}\bar{r}\left[n\right]s^{*}\left[n-\tau_{l}\right]\right\}$$

Because eLoran signals employ BPSK phase coding, the phase observation ${\hat{\Phi }}_{k}$ directly obtained from the correlation output is a noisy wrapped-phase observation and is affected by the π-ambiguity introduced by BPSK phase reversal. In addition, since the carrier phase is inherently 2π-periodic, a single-epoch phase observation alone cannot uniquely determine the total number of complete carrier cycles experienced during signal propagation. Therefore, an integer-cycle ambiguity is also present. If this ambiguity is not properly constrained, it may appear as a fixed integer-cycle offset in the propagation-delay estimate inferred from the carrier phase. Under signal loss of lock, phase jumps, or unstable frequency-offset estimation, it may also lead to discontinuities in carrier-phase tracking and further degrade carrier-phase-aided TOA estimation.

According to (4), (20) can be equivalently written as [8]

(21) $${\hat{\Phi }}_{k}={\mathrm{wrap}}_{2\pi }\left(-2\pi f_{c}\tau_{k}+\phi_{0}\left(k\right)+m_{k}\pi \right)+v_{\phi ,k}$$

where $wrap_{2\pi }\;\left(\cdot \right)$ maps the phase into the interval (−π, π], $m_{k}\pi$ denotes the π-ambiguity term introduced by BPSK phase coding, and $v_{\phi ,k}$ is the phase-observation noise. Further considering the periodic nature of the carrier phase, the complete carrier phase can be expressed as [20]

(22) $$\Phi_{k}={\hat{\Phi }}_{k}+2\pi N_{k}$$

where $N_{k}$ denotes the integer-cycle ambiguity. For a single-epoch carrier-phase observation, $N_{k}$ cannot be determined from the phase observation alone. The TOA measurement $\tau_{k}^{\left(m\right)}$ obtained from (16) provides an absolute propagation-delay reference, which can be used to constrain the feasible integer-cycle range corresponding to the carrier phase [21]. Meanwhile, the state prediction and smoothing effect of the Kalman filter suppress incorrect integer-cycle selection caused by noise, phase wrapping, or short-term disturbances. Therefore, provided that the initial TOA estimate is reliable and no long-term loss of lock occurs, the integer-cycle ambiguity does not accumulate continuously; instead, it is constrained within a reasonable range by the TOA reference and filter prediction.

Based on the TOA measurement $\tau_{k}^{\left(m\right)}$, the propagation-phase reference term $-2\pi f_{c}\tau_{k}^{\left(m\right)}$ can first be computed. It is then combined with the predicted phase state to perform BPSK phase-reversal decision, phase unwrapping, and integer-cycle constraint. The baseband phase estimate can thus be written as

(23) $${\hat{\phi }}_{0}\left(k\right)={\;\mathrm{wrap}}_{2\pi }\left(-2\pi f_{c}\tau_{k}^{\left(m\right)}-m_{k}\pi \right)$$

In the recursive implementation, the phase unwrapping of ${\hat{\phi }}_{0}\left(k\right)$ is determined jointly by the predicted phase from the previous epoch, the frequency-offset state, and the current TOA observation. In summary, the proposed method does not rely on the carrier phase alone to recover the absolute propagation range. Instead, it uses the absolute delay reference provided by TOA to constrain the carrier phase and employs recursive filtering to reduce the effect of incorrect integer-cycle selection. Consequently, in this framework, the integer-cycle ambiguity mainly affects the phase-unwrapping and integer-cycle decision process, and its influence can be effectively mitigated by the TOA constraint and filter prediction.

However, the absolute TOA measurement by itself cannot resolve the integer-cycle ambiguity of the carrier phase. It only provides a coarse but absolute delay reference to bound the feasible integer-cycle candidates. For a carrier frequency of $f_{c}=100\;\mathrm{kHz}$, the carrier period is $T_{c}=1/f_{c}=10\;\mu s$. Therefore, two adjacent integer-cycle hypotheses correspond to a propagation-delay difference of 10 μs. In the recursive framework proposed in this paper, the integer cycle ${\hat{N}}_{k}$ is selected by comparing the propagation-delay candidates inferred from the carrier phase with both the TOA measurement and the predicted delay state from the Kalman filter:

(24) $${\hat{N}}_{k}=\underset{N_{K}}{\arg\;\min}\left[\frac{{\left(\tau_{\phi ,\;k}^{\left(N\right)}-\tau_{k}^{\left(m\right)}\right)}^{2}}{\sigma_{TOA}^{2}}+\frac{{\left(\tau_{\phi ,\;k}^{\left(N\right)}-\tau_{k\;|\;k-1}\right)}^{2}}{\sigma_{Pred}^{2}}\right]$$

where $\tau_{\phi ,\;k}^{\left(N\right)}$ denotes the propagation delay inferred from the unwrapped carrier phase under the integer-cycle hypothesis $N_{K}$, $\tau_{k}^{\left(m\right)}$ is the TOA measurement with uncertainty $\sigma_{TOA}^{2}$ representing the variance of the TOA measurement, and $\tau_{k\;|\;k-1}$ is the predicted delay from the Kalman filter with uncertainty $\sigma_{Pred}^{2}$ representing the variance of the predicted delay. The ambiguity can be reliably bounded when the combined TOA and prediction error is smaller than approximately half a carrier cycle:

(25) $$\left|\tau_{k}^{\left(m\right)}-\tau_{k\;|\;k-1}\right|<\frac{T_{c}}{2}=5\;\mu s$$

Under this condition, the correct integer-cycle candidate is separated from the adjacent candidates by more than the effective uncertainty of the coarse delay reference. However, when the TOA error, phase disturbance, or prediction error becomes comparable to or larger than half a carrier cycle, the proposed method cannot guarantee unique integer-cycle resolution. In such cases, an incorrect cycle decision may introduce a step-like delay error with an interval of 10 μs. Therefore, in the present work, the carrier phase is used mainly to smooth and refine the TOA estimate after coarse TOA-based cycle bounding, rather than to independently recover the absolute propagation delay.

### 4.2. Dynamic Characteristics of Carrier Phase and TOA

Assume that the carrier component in the received eLoran signal $x\left(t\right)$ has a spectral center frequency $f_{c}$, with an equivalent initial phase $\phi_{0}$, and that an equivalent frequency offset Δf exists due to receiver oscillator instability, motion-induced Doppler, or slow propagation variations. From (4), the carrier phase can be expressed as

(26) $$\Phi_{k}\left(t\right)=2\pi \left(f_{c}+\Delta f\right)t+\phi_{0}$$

Its first- and second-order time derivatives are

(27) $$\dot{\Phi }\left(t\right)=\omega \left(t\right)=2\pi \left[f_{c}+\Delta f\right]$$

(28) $$\ddot{\Phi }\left(t\right)=2\pi \frac{d\Delta f}{dt}$$

On an epoch scale, the carrier-phase dynamics can be approximately described by

(29) $$\phi_{0}\left(k+1\right)=\phi_{0}\left(k\right)+2\pi \Delta f_{k}T_{G}+w_{\phi ,k}=\phi_{0}\left(k\right)+\omega_{k}T_{G}+w_{\phi ,k}$$

(30) $$\omega_{k+1}=\omega_{k}+w_{\omega ,k}$$

where $w_{\phi ,k}$ denotes the random phase perturbation, and $w_{\omega ,k}$ denotes the random-walk term of frequency. To describe the deterministic carrier-phase dynamics, the observation noise term is temporarily omitted in this subsection and will be incorporated later in the measurement-noise model.

Therefore, when establishing a carrier-phase tracking model for eLoran, the state vector $x_{k}$ should at least include the baseband carrier phase $\phi_{k}$ and its first-order rate $\dot{\Phi }\left(k\right)$ (i.e., the equivalent frequency offset $\omega_{k}$). To additionally impose a smoothing constraint on the long-term evolution of the carrier phase, the TOA delay $\tau_{k}$ and its drift rate ${\dot{\tau }}_{k}$ (i.e., an equivalent drift) are further introduced [22,23]

(31) $$\tau_{k+1}=\tau_{k}+{\dot{\tau }}_{k}T_{G}+w_{\tau ,k}$$

(32) $${\dot{\tau }}_{k+1}={\dot{\tau }}_{k}+w_{\dot{\tau },k}$$

(33) $$x_{k}={\left[\tau_{k},\;{\dot{\tau }}_{k},\;\phi_{k},{\dot{\phi }}_{k}\right]}^{T}$$

where ${\dot{\phi }}_{k}$ represents the residual baseband frequency offset after accounting for the delay-induced phase rate, rather than a duplicate representation of the TOA drift rate ${\dot{\tau }}_{k}$.

### 4.3. State-Space Model

For a continuous-time system, the following general state-space model can be used:

(34) $$\dot{x}\left(t\right)\;=\;Fx\left(t\right)\;+\;Ge\left(t\right)$$

(35) $$z\left(t\right)=Cx\left(t\right)+v\left(t\right)$$

where F denotes the continuous-time state-transition matrix describing the dynamics of the system state vector $x\left(t\right)$, and G maps the process-noise vector $e\left(t\right)$ into the state dynamics. The measurement vector $z\left(t\right)$ is related to the system state through the measurement matrix C, while $v\left(t\right)$ represents the measurement-noise vector. On an epoch scale, discretizing the above continuous model yields [6]

(36) $$x_{k}=A_{k}x_{k-1}+q_{k-1}$$

(37) $$Z_{k}=Hx_{k}+v_{k}$$

where $A_{k}$ and H denote the discrete-time state-transition matrix and observation matrix, respectively, mapping the previous state $x_{k-1}$ to the current state $x_{k}$ and the state $x_{k}$ to the discrete observation $Z_{k}$. The process noise and measurement noise are represented by $q_{k-1}$ and $v_{k}$, respectively.

### 4.4. State Equations and Covariance Matrices of the Joint Carrier-Phase Model

Based on (33), define the noise vector as [13,24]

(38) $$e\left(t\right)={\left[e_{\tau }\left(t\right),\;e_{\dot{\tau }}\left(t\right),\;e_{\phi }\left(t\right),\;e_{\dot{\phi }}\left(t\right)\right]}^{T}$$

The state vector is regarded as the combination of a TOA subsystem and a carrier-phase subsystem [25]:

(39) $${\left[\tau_{k},\;{\dot{\tau }}_{k}\;\right]}^{T}\;=\;\left[\begin{array}{cc} 1 & T_{G}\\ 0 & 1 \end{array}\right]{\left[\tau_{k-1},\;{\dot{\tau }}_{k-1}\;\right]}^{T}\;+\;q_{k}^{\left(\tau \right)}$$

(40) $${\left[\phi_{k},\;{\dot{\phi }}_{k}\;\right]}^{T}=\left[\begin{array}{cc} 1 & T_{G}\\ 0 & 1 \end{array}\right]{\left[\phi_{k-1},\;{\dot{\phi }}_{k-1}\;\right]}^{T}+q_{k}^{\left(\phi \right)}$$

Combining them gives the state transition matrix F:

(41) $$F\;=\;\left[\begin{array}{cccc} 1 & T_{G} & 0 & 0\\ 0 & 1 & 0 & 0\\ 0 & 0 & 1 & T_{G}\\ 0 & 0 & 0 & 1 \end{array}\right]$$

This matrix is obtained by combining two first-order constant-velocity state-evolution models. The upper-left 2 × 2 block describes the TOA and TOA-drift subsystem, while the lower-right 2 × 2 block describes the carrier-phase and phase-rate subsystem [5,13].

Assume the noise components are mutually independent, zero-mean white noises [26]. Their power spectral densities include four components: TOA measurement noise $S_{\tau }$, TOA drift random-walk noise $S_{\dot{\tau }}$, carrier phase noise $S_{\phi }$, and carrier frequency noise $S_{\dot{\phi }}$. Then

(42) $$S_{e}=E\left\{e\left(t\right)e^{T}\left(t\right)\right\}=\mathrm{diag}\left(S_{\tau },S_{\dot{\tau }},S_{\phi },S_{\dot{\phi }}\right)$$

where $S_{e}$ denotes the power spectral density matrix of the continuous-time process-noise vector $e\left(t\right)$. The vector $e\left(t\right)$ represents the process perturbations acting on the TOA, TOA drift rate, carrier phase, and carrier-phase rate states. Therefore, the diagonal elements of $S_{e}$ describe the corresponding process-noise intensities. They should not be interpreted as measurement-noise variances. The measurement-noise covariance matrix is defined separately in the measurement model.

Let the state update step be $T_{s}$, which equals the epoch interval $T_{G}$. After discretization, the state transition matrix becomes

(43) $$A_{k}=e^{FT_{s}}=\left[\begin{array}{cccc} 1 & T_{s} & 0 & 0\\ 0 & 1 & 0 & 0\\ 0 & 0 & 1 & T_{s}\\ 0 & 0 & 0 & 1 \end{array}\right]$$

Thus, the discrete state equation of the joint TOA–carrier-phase Kalman filter model is

(44) $$x_{k}=A_{k}x_{k-1}+w_{k-1}$$

where $w_{k-1}$ is the process-noise vector induced by integrating $e\left(t\right)$. Through integration over one sampling interval, the continuous-time process noise is converted into the discrete-time process-noise covariance matrix $Q_{k}$ [6]:

(45) $$Q_{k}\;=\;\int_{0}^{T_{s}}A\left(\tau \right)GS_{e}G^{T}A^{T}\left(\tau \right)d\tau$$

And:

(46) $$A\left(\tau \right)\;=\;\left[\begin{array}{cccc} 1 & \tau & 0 & 0\\ 0 & 1 & 0 & 0\\ 0 & 0 & 1 & \tau \\ 0 & 0 & 0 & 1 \end{array}\right]$$

Since the TOA subsystem and the carrier-phase subsystem are mutually independent, $Q_{k}$ can be written in a block-diagonal form:

(47) $$Q_{k}\;=\;\left[\begin{array}{cc} Q_{\tau } & 0\\ 0 & Q_{\phi } \end{array}\right]$$

with

(48) $$Q_{\tau }=\left[\begin{array}{cc} S_{\tau }T_{s}+\frac{S_{\dot{\tau }}T_{s}^{3}}{3} & \frac{S_{\dot{\tau }}T_{s}^{2}}{2}\\ \frac{S_{\dot{\tau }}T_{s}^{2}}{2} & S_{\dot{\tau }}T_{s} \end{array}\right]$$

(49) $$Q_{\phi }=\left[\begin{array}{cc} S_{\phi }T_{s}+\frac{S_{\dot{\phi }}T_{s}^{3}}{3} & \frac{S_{\dot{\phi }}T_{s}^{2}}{2}\\ \frac{S_{\dot{\phi }}T_{s}^{2}}{2} & S_{\dot{\phi }}T_{s} \end{array}\right]$$

The measurement-noise model should include the obtained TOA measurement $\tau_{k}^{\left(m\right)}$ and the carrier-phase observation $\phi_{k}^{\left(m\right)}$ derived from I/Q processing. From (3) and (19), the TOA measurement can be expressed as [6]

(50) $$\tau_{k}^{\left(m\right)}=\tau_{k}+v_{k}$$

The measurement equation is

(51) $$z_{k}={\left[\tau_{k}^{\left(m\right)},\;\phi_{k}^{\left(m\right)}\right]}^{T}=Hx_{k}+v_{k}$$

The covariance matrix of the measurement noise is [27,28]

(52) $$R=\left\{v_{k}v_{k}^{T}\right\}=\left[\begin{array}{cc} \sigma_{\tau }^{2} & 0\\ 0 & \sigma_{\phi }^{2} \end{array}\right]$$

where $\sigma_{\tau }^{2}$ is the TOA measurement jitter, which is related to the SNR and the pulse width; $\sigma_{\phi }^{2}$ is the phase discriminator noise variance, which is approximately proportional to 1/SNR.

## 5. Kalman Filter Model Establishment

### 5.1. Noise and Channel Errors

In Section 3 of this study, the epoch folding analysis approximates the noise as independent and identically distributed (i.i.d.) in order to illustrate the SNR gain mechanism under ideal conditions. Under this assumption, multi-epoch averaging can effectively suppress noise while achieving coherent accumulation of signal energy. However, in practical eLoran reception, the random noise after front-end filtering may contain residual narrowband interference and band-limited correlated components, and therefore does not strictly satisfy the i.i.d. assumption [26]. To better reflect realistic conditions, this study introduces a first-order autoregressive (AR) correlated noise model to simulate interference with stronger temporal correlation.

It is important to emphasize that these two assumptions are not contradictory, but correspond to different levels of analysis: the i.i.d. assumption is used to illustrate the theoretical gain of epoch folding under ideal or weakly correlated noise, whereas the AR-based model in this section is employed to evaluate the robustness of the method under more complex and non-ideal conditions. When the correlation time of the noise is short relative to the GRI interval, or the correlation between different epochs is weak, epoch folding can still achieve effective SNR improvement, although the gain is slightly lower than in the ideal i.i.d. case [29].

In an eLoran receiver, the raw signal collected by the antenna typically contains multiple noise and interference components. Therefore, the receiver front end commonly employs an analog bandpass filter and a digital FIR filter to suppress out-of-band interference and improve the SNR. After filtering, the dominant equivalent noise terms that affect the observations can be categorized into three types: random noise, impulsive noise, and slowly varying multipath error [2].

In carrier or baseband phase estimation, the accumulation of random noise also increases the uncertainty of the equivalent frequency bias.

For occasional strong burst interference, this paper models impulsive noise using a Poisson shot-noise (Poisson arrival) impulsive-noise model, where impulsive events on the time axis follow a Poisson process. With an arrival rate λ, each impulsive burst is represented as a carrier waveform with an exponentially decaying envelope [30]:

(53) $$n_{i}\left(t\right)\;=\;A\exp\left(-\frac{t-t_{0}}{\tau_{0}}\right)\cos\left(2\pi f_{c}t\;+\;\theta \right),\;\;\;t\geq t_{0}$$

where A is the burst amplitude, $\tau_{0}$ is the decay time constant, and θ is a random initial phase. Impulsive noise usually manifests as outlier measurements in TOA and phase estimation rather than a persistent bias. Based on this property, instead of directly enlarging the overall measurement-noise variance in the subsequent joint Kalman filter, an adaptive measurement-noise inflation mechanism is introduced to suppress outlier observations, thereby improving filter stability under impulsive-noise conditions.

A multipath component has an amplitude, delay, and phase that are independent relative to the direct-path component. The received signal can be expressed as [9]

(54) $$x\left(t\right)=s\left(t-\tau \right)+a_{m}s\left(t-\tau -\Delta \tau_{m}\right)\cos\left(2\pi f_{c}t+\phi +\phi_{m}\right)$$

where $a_{m}$ is the multipath amplitude ratio, $\Delta \tau_{m}$ is the extra multipath delay, and $\phi_{m}$ is the multipath phase offset. To reflect slow environmental changes, these multipath parameters are modeled as random walks [9,31]:

(55) $$\left\{\begin{array}{cc} a_{m}\left[k\right]= & a_{m}\left[k-1\right]+w_{\alpha }\left[k\right]\\ \Delta \tau_{m}\left[k\right]= & \Delta \tau_{m}\left[k\;-\;1\right]\;+w_{\tau }\left[k\right]\\ \phi_{m}\left[k\right]= & \phi_{m}\left[k\;-\;1\right]\;+\;w_{\phi }\left[k\right] \end{array}\right.$$

where $w_{\alpha }$, $w_{\tau }$, and $w_{\phi }$ are zero-mean Gaussian perturbations. Consequently, multipath yields an equivalent slowly varying delay bias in TOA estimation and additional phase noise in the phase observation.

The above three noise terms differ significantly in statistical characteristics and time scales. Therefore, their impacts on estimation are explicitly mapped into the Kalman filtering framework: random background noise and slow multipath error are mainly reflected through the process-noise covariance Q to describe uncertainty in state evolution; instantaneous observation errors caused by random noise and impulsive noise are represented through the measurement-noise covariance R, while outlier observations induced by impulsive noise are further suppressed via an adaptive inflation strategy. With this modeling approach, the filter can achieve robust joint estimation of TOA, baseband phase, and frequency offset under the coexistence of strong noise, multipath, and burst interference.

### 5.2. Filter Covariance Matrix Setting

For the selection of noise power spectral density parameters, autocorrelation-based estimation may be applied to the noise signals. Considering that ${\dot{\tau }}_{k}$ and ${\dot{\phi }}_{k}$ vary slowly on the epoch scale, for a general receiver the TOA subsystem and the carrier-phase subsystem can be modeled by a constant-velocity (CV) model. From (47)–(49), the process-noise covariance matrices can be simplified as

(56) $$Q_{\tau }=q_{\tau }\left[\begin{array}{cc} \frac{T_{s}^{3}\;}{3} & \frac{T_{s}^{2}}{2}\\ \frac{T_{s}^{2}}{2} & T_{s} \end{array}\right]$$

(57) $$Q_{\phi }=q_{\omega }\left[\begin{array}{cc} \frac{T_{s}^{3}}{3} & \frac{T_{s}^{2}}{2}\\ \;\frac{T_{s}^{2}}{2} & T_{s} \end{array}\right]$$

(58) $$Q=\left[\begin{array}{cc} Q_{\tau } & 0\\ 0 & Q_{\phi } \end{array}\right]$$

where $q_{\tau }$ and $q_{\omega }$ are the discrete-time parameters corresponding to the continuous-time random-process intensities $S_{\dot{\tau }}$ and $S_{\dot{\phi }}$, respectively. The measurement-noise covariance matrix R is the same as in (52).

### 5.3. Standard KF Recursion

Under the state-space model described above, the standard discrete Kalman filter recursion consists of prediction and update steps. The state and covariance prediction are [25]

(59) $${\hat{x}}_{k|k-1}=F{\hat{x}}_{k-1|k-1}$$

(60) $$P_{k|k-1}=FP_{k-1|k-1}F^{T}+Q$$

The Kalman gain is

(61) $$K_{k}=P_{k-1|k-1}H^{T}{\left(HP_{k|k-1}H^{T}+R\right)}^{-1}$$

The state and covariance update are

(62) $${\hat{x}}_{k|k}={\hat{x}}_{k|k-1}+K_{k}\left(z_{k}-H{\hat{x}}_{k|k-1}\right)$$

(63) $$P_{k|k}=\left(I-K_{k}H\right)P_{k|k-1}$$

By incorporating the baseband phase observation $\phi_{k}^{\left(m\right)}$ into the Kalman filter recursion, a smooth and continuous baseband carrier-phase state can be obtained.

### 5.4. MMA-KF Design and Implementation

In practical applications, due to factors such as propagation-path perturbations, environmental noise variations, strong burst interference, and receiver oscillator stability, the system dynamics and noise statistics are often difficult to model accurately, and the filter parameters may vary within a reasonable range. To enhance adaptability under complex conditions, this paper introduces a multiple-model adaptive Kalman filter (MMA-KF) to perform joint state estimation [7]. The MMA-KF consists of MM parallel sub-Kalman filters. Each sub-filter corresponds to a different pair of process-noise covariance $Q^{\left(i\right)}$ and measurement-noise covariance $R^{\left(i\right)}$, representing possible models under different dynamics and observation uncertainties [32].

Based on a set of baseline model parameters $\left\{Q^{0},R^{0}\right\}$ established in Section 3, a parallel model set is constructed around the baseline model $M^{0}$. The remaining models are obtained by scaling the covariance matrices:

(64) $$Q^{i}\;=\;\alpha_{i}Q^{0},\;\;\;R^{i}\;=\;\beta_{i}R^{0},\;\;\;i\;=\;1,\ldots ,M-1$$

where $Q^{0}$ and $R^{0}$ are set with reference to Equations (39)–(46) for initializing the filter. During the initial filtering steps, they play a key role in properly weighting the predicted states and the observations, and their values can be determined based on prior system knowledge or historical data. $\alpha_{i}$ and $\beta_{i}$ are perturbation factors. Since the baseline model is assigned a higher prior credibility, it is given a larger initial weight:

(65) $$\mu_{0}^{0}\;=\;\mu_{\max},\;\;\;\mu_{0}^{i}\;=\;\frac{1\;-\;\mu_{\max}}{M\;-\;1}$$

where $\mu_{max}\;\in$ (0, 1) is the prior confidence coefficient.

For the i-th sub-filter, using (59)–(63), the innovation and its covariance are computed as [7]

(66) $$v_{k}^{\left(i\right)}=Z_{k}-{H{\hat{x}}^{\left(i\right)}}_{k|k-1}$$

(67) $$S_{k}^{\left(i\right)}=HP_{k|k-1}^{\left(i\right)}H^{T}+R^{\left(i\right)}$$

where $v_{k}^{\left(i\right)}$ denotes the innovation (or measurement residual) of the i-th sub-filter at time step k, $S_{k}^{\left(i\right)}$ represents the corresponding innovation covariance matrix, calculated based on the predicted state covariance and the measurement-noise covariance $R^{\left(i\right)}$.

Under the Gaussian innovation assumption, the likelihood function of the ii-th model is

(68) $$\Lambda_{k}^{\left(i\right)}=\frac{1}{\sqrt{{\left(2\pi \right)}^{n_{z}}\left|S_{k}^{\left(i\right)}\right|}\;}\exp\left[-\frac{1}{2}v_{k}^{\left(i\right)}S_{k}^{\left(i\right)-1}v_{k}^{\left(i\right)}\right]$$

where $n_{z}$ denotes the dimension of the measurement vector $z_{k}$. In this paper, $z_{k}$ consists of the TOA measurement and the carrier-phase measurement; therefore, $n_{z}=2$.

Because $\Lambda_{k}^{\left(i\right)}$ can be extremely small, direct computation may suffer from underflow (e.g., $\exp\left(\cdot \right)\to 0$) in finite-precision arithmetic. Therefore, the log-likelihood is used for numerically stable evaluation:

(69) $$l_{k}^{\left(i\right)}\;≜\log\Lambda_{k}^{\left(i\right)}\;=\;-\frac{1}{2}\left(n_{z}\log\left(2\pi \right)\;+\;\log\left|S_{k}^{\left(i\right)-1}\right|\;+\;v_{k}^{\left(i\right)}S_{k}^{\left(i\right)-1}v_{k}^{\left(i\right)}\right)$$

The model weights are updated recursively according to Bayes’ rule [33]:

(70) $${\bar{l}}_{k}^{\left(i\right)}\;=\;l_{k}^{\left(i\right)}\;-\;\underset{j}{\max}l_{k}^{\left(j\right)}$$

(71) $$\mu_{k}^{\left(i\right)}=\frac{\exp\left({\bar{l}}_{k}^{\left(i\right)}\mu_{k-1}^{\left(i\right)}\right)}{\sum_{j=1}^{M}\exp\left({\bar{l}}_{k}^{\left(j\right)}\mu_{k-1}^{\left(j\right)}\right)}$$

Finally, the MMA-KF fused state estimate is obtained by the weighted combination of sub-filter estimates:

(72) $$x_{k}^{\mathrm{MMA}}\;=\;\sum_{j\;=\;1}^{M}\mu_{k}^{\left(j\right)}{{\hat{x}}^{\left(j\right)}}_{k|k}$$

The above derivation generally assumes that the process noise and the measurement noise are both zero-mean Gaussian. In real systems, impulsive noise may cause instantaneous severe failures in correlation detection and phase measurement. Although such events may not have a large variance in a statistical sense, the corresponding measurements can become completely unreliable. From (62), the filter may force the state toward these erroneous measurements, leading to TOA jumps and even filter divergence. Therefore, an adaptive inflation strategy is introduced to suppress such effects:

(73) $$R_{k}\;=\;\left\{\begin{array}{c} R_{k},\;\;\;\left|v_{k}\right|\leq K\sigma \\ \gamma R_{k},\;\;\;\left|v_{k}\right|>K\sigma \end{array}\right.$$

where the standard deviation $\sigma =\sqrt{S_{k}}$ is calculated from the theoretical innovation covariance, for a scalar residual, with [34]

(74) $$\;S_{k}=HP_{k|k-1}H^{T}+R_{k}$$

Therefore, σ is derived from the assumed measurement-noise variance and the predicted state covariance propagated by the Kalman filter, rather than being statistically estimated from residual samples. The coefficient K is the gating threshold coefficient. In this study, it is selected according to the conventional Kσ consistency test; for example, K=3 corresponds approximately to a three-standard-deviation gate under the Gaussian residual assumption. The parameter γ>1 is an empirical covariance-inflation factor. Once the innovation exceeds the gate, the measurement covariance is inflated to $\gamma R_{k}$, thereby reducing the Kalman gain and suppressing the influence of abnormal measurements [35]. The value of γ is selected through simulation-based sensitivity analysis to balance robustness against impulsive disturbances and tracking accuracy under nominal noise conditions.

This strategy can theoretically improve adaptability to system uncertainty. However, in practical conditions—especially when the dynamics are moderate and model mismatch is limited—the carrier-phase tracking performance may not necessarily be significantly better than that of a single Kalman filter [32]. This is because the process-noise and measurement-noise parameters used by a single filter may already characterize the true system dynamics reasonably well. In such cases, adding multiple sub-models with different noise parameters can introduce an averaging effect during fusion, making the estimate slightly smoother over short time scales and not necessarily advantageous in the mean-square-error sense. The primary advantage of MMA-KF is manifested in complex environments where system dynamics and noise characteristics vary significantly over time or are difficult to model accurately; its value lies in improving overall robustness under model uncertainty.

## 6. Results and Discussion

### 6.1. Simulation of eLoran Signal Generation

As described in Section 2, the waveforms of different eLoran signals are nearly identical and are generated by the fixed mathematical function shown in Equation (2). Therefore, by setting a specific GRI, it is straightforward to simulate realistic waveforms; in contrast, simulating real-world noise interference requires consideration of more complex factors.

In Section 5, we systematically modeled noise. By introducing random white noise, correlated band-limited noise, pulse noise, and slowly varying multipath errors, we simulated the various noise interferences that may occur during signal propagation and reception. These include environmental background noise in low-frequency bands, receiver front-end thermal noise, transient disturbances such as lightning discharges or motor switching, and long-term deviations caused by signal reflections and slow environmental changes. This modeling covers the majority of realistic noise effects.

**Figure 3:**
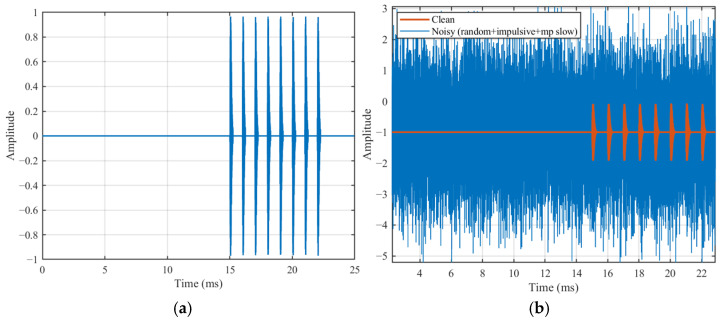
Simulated eLoran Pulse Group. (**a**) Raw eLoran signal. (**b**) Simulated signal after noise addition.

The simulated eLoran signal pulse group is shown in Figure 3a. Each pulse group is configured to contain eight pulses, with an interval of 1 ms between successive pulses. The aforementioned three types of noise are then superimposed onto the original pulses. By adjusting the overall signal-to-noise ratio SNR to −10 dB, as shown in Figure 3b, the eLoran pulses are almost completely buried in noise.

### 6.2. Signal Processing, GRI Search, and TOA Estimation

**Figure 4:**
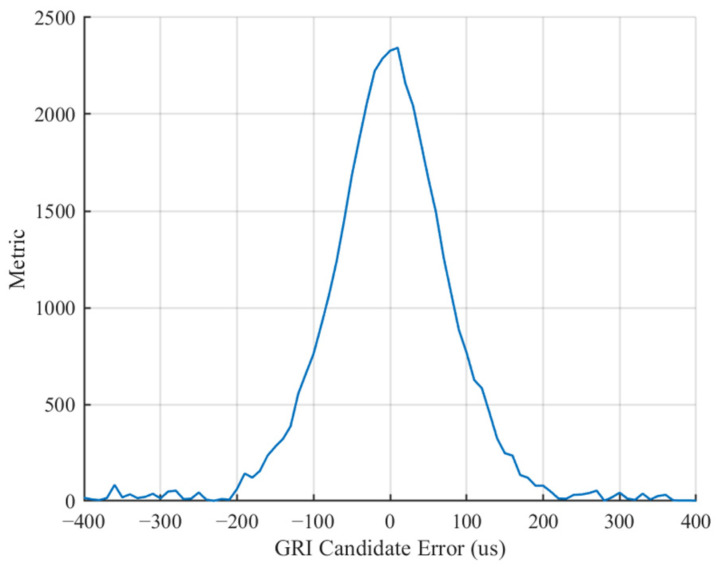
GRI Candidate Error.

By correlating the candidate GRI set with the preset GRI, a unique GRI can be determined from the correlation peak. In practical reception, GRI measurements may be affected by noise, multipath effects, slow drifts in transmission or propagation, and receiver or environmental motion, which typically induce small fluctuations in the GRI. In this chapter, the aforementioned effects are simulated using the previously established noise model and dynamic drift. As shown in Figure 4, the correlation peak exhibits a small error of approximately 1 μs; however, since this error is far smaller than half of the GRI resolution (5 μs), the GRI can still be successfully identified.

**Figure 5:**
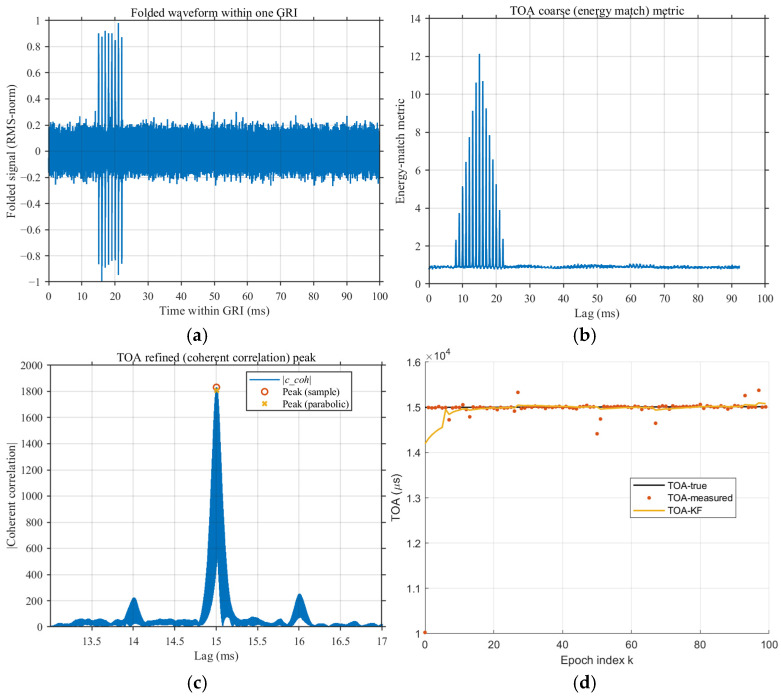
Results of the three-stage TOA processing. (**a**) Signal after GRI−based epoch folding. (**b**) energy-matching metric for coarse TOA estimation. (**c**) TOA correlation peak after refinement and interpolation. (**d**) TOA tracking over epochs.

After successfully identifying the current GRI and performing epoch folding, a comparison between Figure 3b and Figure 5a and shows that the eLoran pulse groups previously buried in noise are effectively aligned and coherently accumulated along the time axis, forming a distinct comb-like peak cluster around 2–20 ms. This region corresponds to the true TOA of the pulse group, and its amplitude is significantly higher than the background noise in other time intervals. The spacing between adjacent peaks is approximately 1 ms, resulting from the energy accumulation of multiple pulses within a single eLoran pulse group. Outside the main peak region, the folded signal is dominated by zero-mean random noise, indicating that epoch folding can effectively improve the SNR without introducing coherent bias.

At the same time, Figure 5a shows that even when using the AR model, the signal SNR after epoch folding is still significantly enhanced. This further validates the viewpoint presented in Section 5.1 regarding noise and channel-error modeling: in the presence of colored noise, epoch folding can still achieve effective coherent accumulation, although the noise suppression effect may be influenced by temporal correlation.

In this study, the initial TOA is set to 15 ms, with microsecond-level drift introduced, and the sampling interval $T_{s}\;=\;1$, consistent with the conditions shown in Figure 5a. As illustrated in Figure 5b, the coarse TOA estimate selects the maximum value within the peak cluster, which is approximately 15 ms.

Since pseudo-correlation peaks may arise between different stations within the same eLoran chain, we take three stations of the Beihai chain in China as an example.

As shown in Table 1, the distances between these stations range from 700 to 1500 km, corresponding to TOA delays exceeding 2.5 ms. Based on Equations (9) and (10), a window threshold of 1.5 ms can be set here, combined with a maximum-value search, to effectively eliminate pseudo-correlation peaks.

In the subsequent refinement process, the TOA accuracy can be improved to the sub-sample level (within ± 0.5 ms). As illustrated in Figure 5c, the curve near the correlation peak exhibits a smooth and sharp peak shape, which can be used to initialize the filter and significantly enhance the stability of subsequent tracking.

With $\mu_{max}$ = 0.5, $\sigma_{\omega }$ = 0.12, and SNR = −10 dB, the convergence speed is defined as:

(75) $$v_{con}\;=\;\frac{1}{t_{con}}\;=\;\frac{1}{\left(kconv\;-\;1\right)\cdot \mathrm{GRI}}$$

where $v_{con}$ is the TOA convergence rate, representing the effective update frequency at which the TOA reaches the predefined convergence threshold. $t_{con}$ is the convergence time and conv is the number of epochs required to achieve the specified convergence threshold.

As shown in Figure 5d, when the convergence threshold is set to 10 μs and the epoch interval is set to 99.6 ms, TOA convergence is completed within six epochs, corresponding to approximately 0.598 s. Due to the impact of impulsive noise, abrupt jumps occasionally appear in the raw TOA measurements. However, the Kalman filter effectively suppresses these outliers through covariance inflation and smoothing, keeping the output stably close to the ground truth.

### 6.3. Comparison of Tracking Performance Between KF and Costas Loop

This subsection is based on the previously described eLoran signal modeling and the experimental conditions shown in Figure 4. Using the Costas loop as a reference, the tracking performance of the Costas loop is compared with that of the Kalman filter.

In the configuration of the Costas loop, the loop bandwidth is a key parameter affecting tracking performance. As indicated by Equation (1), when the loop bandwidth is too large, the frequency integrator becomes overly sensitive to single-pulse noise, which can easily cause frequency jumps and transient loss of lock; conversely, when the loop bandwidth is too small, the loop response becomes sluggish, potentially failing to track frequency variations in high-dynamic scenarios.

For the Costas loop, within the noise model adopted in this study, the most significant factor affecting tracking performance is the single-pulse noise. This is because, under PRI-level updates, the phase measurement error of each pulse is directly accumulated by the integrator, and the instantaneous amplification can lead to frequency jumps. To ensure a fair comparison in the experiments, an additional low-pass filter was applied at the output of the Costas loop. Experimental results show that this low-pass filter has minimal effect on phase tracking, but significantly improves frequency tracking: it effectively smooths the instantaneous impact on the frequency integrator, reduces frequency jumps, and thereby enhances tracking stability. The specific coefficients of the low-pass filter will be discussed in detail in the subsequent sections.

**Figure 6:**
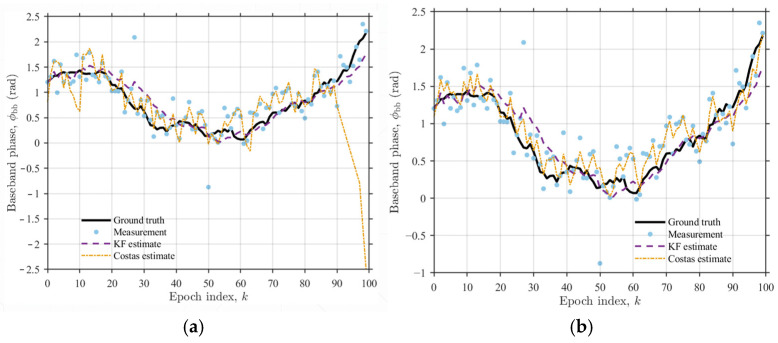
Comparison of baseband phase tracking performance of two methods over 100 epochs. (**a**) *B_L_* = 40 Hz, without low-pass filter. (**b**) *B_L_* = 40 Hz, with low-pass filter.

In terms of phase tracking, the low-pass filter can smooth the phase output, effectively preventing transient loss of lock caused by the accumulation of single-pulse noise. As shown in Figure 6a, when the loop bandwidth is set to 40 Hz and no other filters are applied, the accumulation of single-pulse noise may drive the frequency integrator to its limit, resulting in loss of lock at approximately the 90th epoch. In contrast, the tracking performance in Figure 6b remains stable. However, due to the additional phase lag introduced by the filter, the tracking accuracy may slightly decrease; in this experiment, the impact is approximately 1.8%. It is important to note that even reducing the loop bandwidth cannot completely prevent loss of lock, but can only delay its occurrence. This is because, although a lower bandwidth reduces the loop response speed and slows the rate at which the integrator accumulates to its limit, single-pulse noise will ultimately accumulate and may still cause loss of lock.

To quantify the tracking performance, this study employs the root mean square error (RMSE) metric:

(76) $$RMSE=\sqrt{\frac{1}{K}\sum_{k=1}^{K}{\left(x_{est}\left[k\right]-x_{true}\left[k\right]\right)}^{2}}$$

where $x_{est}\left[k\right]$ denotes the estimated value, $x_{true}\left[k\right]$ represents the corresponding true value, and K is the total number of epochs. By setting the loop bandwidth to 10, 20, 30, and 40 Hz, the RMSE of the Costas loop for phase tracking is obtained, as summarized in the table.

As shown in Table 2 and Figure 6a,b, with the increase in loop bandwidth, the root mean square error (RMSE) of the Costas loop slightly increases, but the variation remains small, maintaining an overall reasonable range of approximately 0.45–0.5 rad. In comparison, the RMSE of the KF is 0.394 rad, which is significantly lower than that of the Costas loop, indicating that the KF exhibits superior phase tracking accuracy.

In terms of frequency tracking, a systematic analysis was conducted on the low-pass filter applied at the output of the Costas loop. This filter can be regarded as a first-order recursive low-pass filter, with the following update equation [36]:

(77) $$\omega_{d}\left[k\right]=\alpha_{\omega }\omega_{d}\left[k\right]+\left(1-\alpha_{\omega }\right)\omega_{d}\left[k-1\right]$$

where $\alpha_{\omega }\in \left(0,1\right]$ denotes the filter coefficient, which balances the smoothing of transient disturbances with the tracking response speed. When $\alpha_{\omega }$ is too small, the filter provides insufficient smoothing, and single-pulse noise may cause instantaneous jumps in the frequency integrator; conversely, when $\alpha_{\omega }$ is too large, the filter responds too slowly and cannot follow frequency variations in a timely manner, thereby affecting dynamic tracking performance.

**Figure 7:**
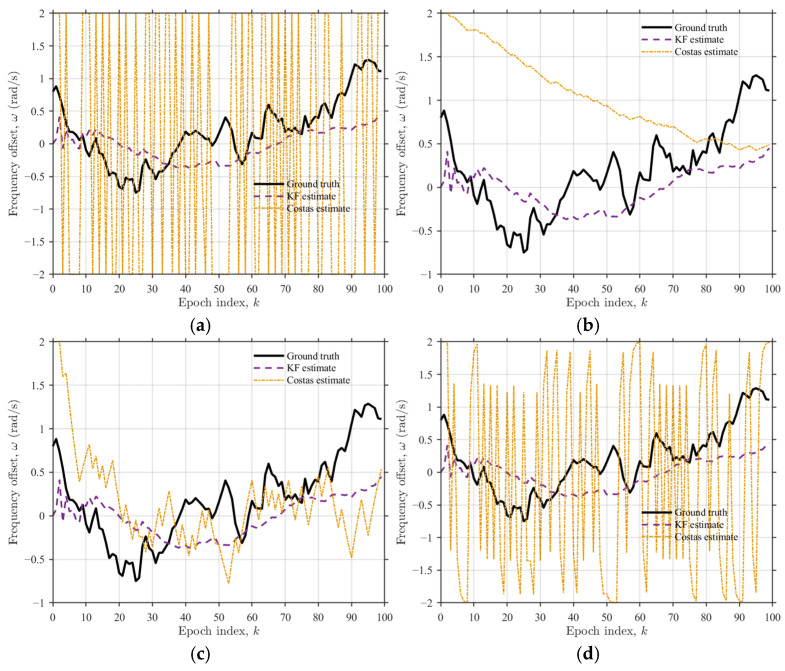
Comparison of frequency tracking performance of two methods over 100 epochs. (**a**) Without low-pass filter. (**b**) With low-pass filter, $\alpha_{\omega }$ = 0.01. (**c**) With low-pass filter, $\alpha_{\omega }$ = 0.1. (**d**) With low-pass filter, $\alpha_{\omega }$ = 0.8.

In the experiment, the low-pass filter coefficient $\alpha_{\omega }$ was scanned over the range of 0.01–1, and the frequency tracking performance of the Costas loop was evaluated using the root mean square error (RMSE). The results are presented in Table 3 and Figure 7.

As shown in Figure 7a–d, when no filter is applied, frequency tracking completely fails; due to the maximum frequency deviation being limited to ±2 rad/s, the frequency continuously jumps within the range [−2, 2] rad/s. When the filter coefficient is too small (e.g., 0.01), the filter provides insufficient smoothing, and the frequency is still affected by single-pulse noise, resulting in degraded performance. In contrast, when the coefficient is set to 0.1, the Costas loop achieves the best frequency tracking performance, with a root mean square error (RMSE) of 0.666 rad/s. However, when the coefficient is too large (e.g., 0.8), the filter responds too slowly, and frequency tracking is essentially lost; when the coefficient is 1.0, the update is effectively frozen, and frequency tracking completely fails. In comparison, the RMSE of the KF is 0.462 rad/s, significantly lower than that of the Costas loop, indicating that under the same low SNR and high-dynamic conditions, the KF provides more robust frequency tracking performance.

### 6.4. Comparison of Tracking Performance Between KF and MMA-KF

In this study, the MMA-KF, as an extension of the standard Kalman filter, is employed to address the issue of reduced convergence speed caused by parameter inaccuracies in a single Kalman filter under extreme conditions.

In the experimental setup, the MMA-KF was configured with 10 parallel filters $A_{1}$, $A_{2}$, …, $A_{10}$, each assigned different initial covariance matrices Q and measurement noise matrices R, as well as distinct initial weights. Among them, $A_{1}$ utilized the optimal parameters of the standard single Kalman filter and was assigned the highest initial weight of 28%, while the remaining filters were initialized along a linear variation, each receiving an equal initial weight of 8%. During operation, the weights of the individual filters were dynamically adjusted based on the comparison between their predicted state values (a priori estimates) and the corresponding measurements (observations), enabling adaptive optimization in response to the current environment.

**Figure 8:**
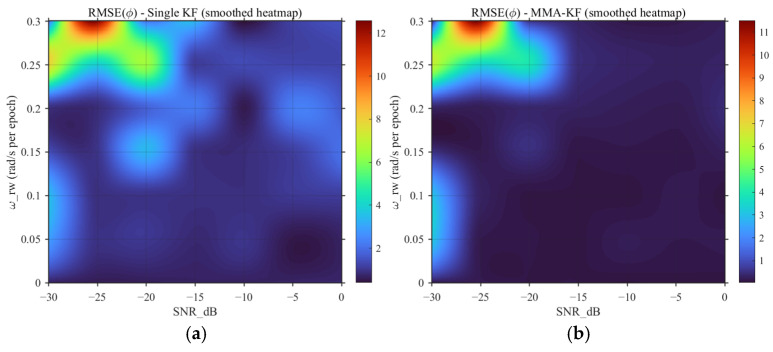
Phase residual heat maps. (**a**) Single KF. (**b**) MMA-KF.

As shown in Figure 8, while keeping the parameters of the original single Kalman filter unchanged, this experiment introduces the aforementioned MMA-KF. A parameter sweep was conducted over the ranges $\sigma_{\omega }\in \left(0,\;0.3\right)$ and SNR $\in \left(-30,\;0\right)$, and heat maps were used to illustrate the normalized residuals (RESM) relative to the true phase values.

It should be noted that the colorbar on the right side of Figure 8 represents the reference scale for the RMSE values. This scale does not start from 0; instead, the KF-RMSE value of 0.3936 under the parameter condition $\sigma_{\omega }$ = 0.12 rad/s and SNR = −10 dB is set as the baseline threshold. When the residual corresponding to a given parameter combination is less than 0.3936, it is considered to have achieved good tracking performance, and its specific value is no longer further distinguished or displayed. This treatment reduces excessive color variations in the low-residual region and makes the performance differences between the two filters over a larger residual range more visually distinguishable. The interval between the baseline threshold value of 0.3936 and the maximum residual value of 12.5645 appearing in the figure is then evenly divided into 10 levels, which are used as the unified colorbar reference range in Figure 8.

By comparing Figure 8a,b, it can be seen that, within the same parameter sweep range, the MMA-KF achieves a smaller phase RMSE and a wider stable operating region than the single Kalman filter (RMSE < 0.3936). For the single KF, as shown in Figure 8a, when $\sigma_{\omega }$ is relatively small or the SNR is relatively high, the residual remains generally low, indicating that the fixed-parameter KF can effectively lock the carrier phase under normal dynamic conditions and favorable SNR levels. In contrast, Figure 8b shows that, under the same parameter conditions, the MMA-KF yields lower residuals and a slightly enlarged stable operating region. This indicates that the parallel multiple-model structure can, through competition and weighted fusion among different Q/R parameter models, allow at least some sub-filters to match the current signal environment, thereby reducing the performance degradation caused by model mismatch in a single fixed-parameter filter.

Overall, both filters can achieve carrier-phase lock under normal parameter conditions. However, the MMA-KF exhibits stronger adaptability and better robustness under low-SNR conditions and larger frequency random-walk intensity. Therefore, the single KF is more suitable for scenarios where the parameters are known and the environment is relatively stable, whereas the MMA-KF is more suitable for practical receiving environments with varying noise statistics and signal dynamics. The main difference between the two methods is reflected not only in the steady-state residual level, but also in the subsequent convergence speed, which is particularly important for carrier-phase tracking applications requiring high precision, strong robustness, and low latency.

Therefore, we further investigated the convergence time of the two filters under different $\sigma_{\omega }$ and SNR conditions using the same calculation method as in Equation (74). In this study, the convergence time is defined as the earliest epoch at which the RMSE gradually becomes stable over time and no longer exhibits obvious fluctuations. To reduce computational cost and facilitate the distinction between different parameter conditions, $\sigma_{\omega }$ and SNR were discretely swept with step sizes of $\Delta \sigma_{\omega }$= 0.01 rad/s and ΔSNR = 1 dB, respectively.

**Figure 9:**
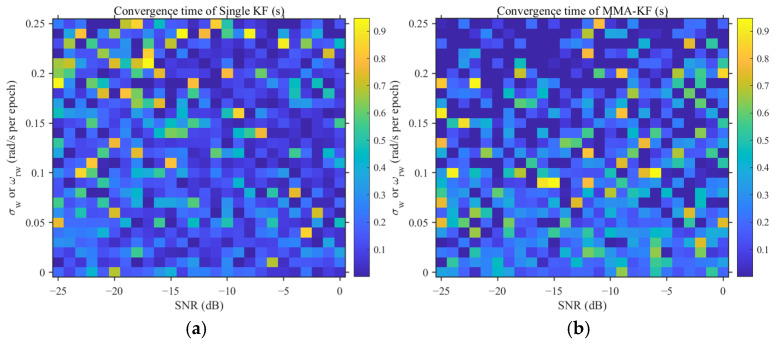
Convergence time heat maps. (**a**) Single KF. (**b**) MMA-KF.

As shown in Figure 9, in the region where the parameter conditions are close to the optimal operating point of the single KF, namely $\sigma_{\omega }$ = 0.12 rad/s and SNR = −20 dB, the single Kalman filter $A_{1}$ exhibits the fastest convergence speed. In contrast, the MMA-KF is affected by the other parallel filters with less accurately matched initial parameters during the early stage of weighted averaging, resulting in a slightly slower overall convergence speed. However, when $\sigma_{\omega }$ increases above 0.1 rad/s and the SNR decreases below −10 dB, the convergence speed of the MMA-KF begins to become significantly faster than that of the single Kalman filter. This indicates that, when the number of parallel filters in the MMA-KF is sufficient and their initial parameter coverage effectively includes the current signal environment, at least one sub-filter can closely match the actual signal state, thereby enabling the MMA-KF to converge faster than a single Kalman filter with fixed parameters.

Based on the differences in convergence time between the two filters under different environmental parameters, a dynamic threshold management mechanism can be introduced according to Equation (70). This mechanism can adaptively select an appropriate filtering strategy according to the current signal environment, thereby effectively reducing unnecessary computational overhead and power consumption in practical applications while approaching the optimal convergence time of the MMA-KF as closely as possible.

**Figure 10:**
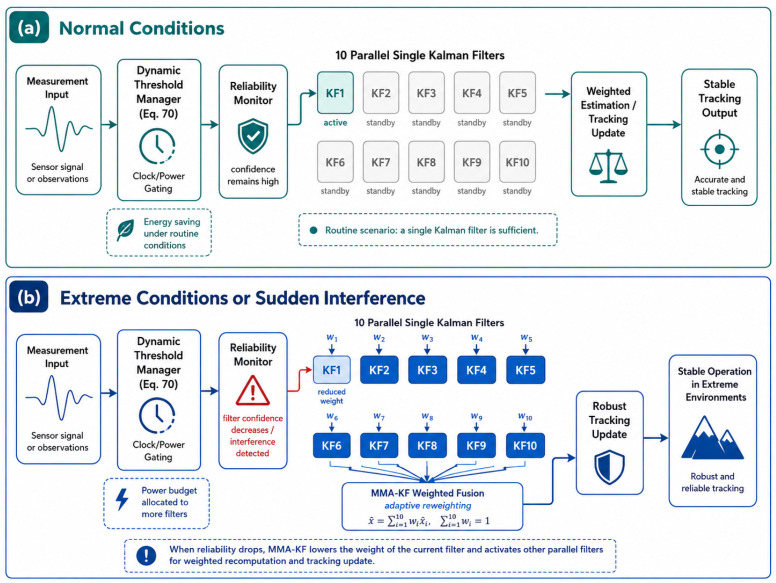
Adaptive MMA-KF Architecture with Gating Management. (**a**) Normal Conditions. (**b**) Extreme Conditions or Sudden Interference.

As shown in Figure 10, under normal conditions, a single Kalman filter modeled based on routine scenarios is sufficient; however, when the system detects a drop in the current filter’s reliability or encounters sudden interference, the MMA-KF automatically reduces the weight of that filter and activates other parallel filters for weighted recomputation and tracking updates, thereby ensuring stable system operation even under extreme conditions.

## 7. Conclusions

This work targets key challenges in eLoran timing reception under complex interference environments characterized by low SNR and moderate-to-high dynamics, where TOA measurements are easily corrupted by noise, carrier-phase tracking is prone to loss of lock, and frequency-offset estimation may exhibit long-term drift. To this end, a joint TOA–carrier-phase measurement and tracking framework consisting of reliable signal acquisition, robust measurement construction, and unified recursive estimation. The central idea is to exploit the complementary representations of the same propagation delay provided by TOA and carrier phase: TOA offers an absolute delay reference, whereas the carrier phase provides high short-term resolution and continuity. Their fusion improves both short-term smoothness and long-term stability, thereby suppressing drift caused by phase ambiguity and accumulated frequency-offset errors and enhancing trackability under weak-signal conditions.

For acquisition and observable construction, an engineering-oriented three-step procedure is designed. First, chain identification is performed and the standard GRI is fixed. Second, epoch folding is applied under the fixed GRI to accumulate signal energy and significantly improve the effective SNR. Third, a two-stage TOA refinement is carried out: a coarse TOA estimate is obtained via energy matching using a non-coded envelope template, then the peak location is refined in its neighborhood via coherent correlation with a coded template, and sub-sample TOA measurement is achieved through local parabolic interpolation around the correlation peak. This provides a stable initialization and high-quality measurements for subsequent tracking. For phase-observable construction, baseband phase measurements are formed via coherent downconversion and accumulation, and BPSK branch selection and phase unwrapping are performed using the predicted reference, mitigating instability due to phase wrapping and coding-induced ambiguity.

For joint tracking, a unified state-space model incorporating TOA bias and drift rate, baseband carrier phase, and frequency offset is established, and Kalman filtering is used to recursively fuse TOA and phase. The TOA observation supplies an absolute constraint that prevents unanchored long-term drift of the phase-integrated term, while the phase observation provides highly sensitive short-term dynamic information and imposes a smoothing constraint on TOA drift, substantially enhancing the continuity of the timing output. To address uncertainty in noise statistics and dynamic models in real environments, as well as outlier measurements induced by impulsive noise, a parallel multiple-model adaptive Kalman filter (MMA-KF) with innovation-likelihood-based adaptive weight updating is introduced, together with measurement-noise covariance inflation to suppress the biasing effect of abnormal measurements on state updates, thereby improving robustness and low latency in complex electromagnetic environments.

Simulation results verify the effectiveness of the proposed method under low SNR and compounded interference. Under the specified convergence criterion, TOA converges within approximately 1 s and remains stable, and faster convergence may be achieved under other parameter settings, while the baseband phase remains continuous and frequency-offset estimation stays stable. Compared with the conventional Costas loop, the Costas loop faces the classic bandwidth–noise trade-off under low SNR and enhanced dynamics; the frequency-offset estimate tends to fluctuate and may develop accumulated bias and trend-like drift in the middle and later stages. In contrast, the joint KF framework, by introducing the absolute TOA reference and jointly modeling phase dynamics, more effectively suppresses long-term drift caused by ambiguity and accumulated frequency-offset errors, providing superior overall robustness.

Future work can proceed along two directions: (i) incorporating real measurement data to further refine the statistical modeling of random noise, impulsive noise, and slowly varying multipath, and exploring adaptive tuning to reduce model mismatch; and (ii) for long-term operation, more tightly integrating sampling-clock bias, small GRI drift, and slow propagation variations into the state modeling to further improve long-term stability and engineering applicability.

## Figures and Tables

**Table 1:** Time Differences of Pseudo-Correlation Peaks for Different Stations within the Same eLoran Chain.

*City*	Distance from Reference	Time Difference
RongCheng	739 km	2.46 ms
XuanCheng	852 km	2.84 ms
HeLong	1500 km	5.00 ms

**Table 2:** RMSE of the Costas Loop with Different Loop Bandwidths.

*B_L_* (Hz)	10	20	30	40
Costas loop	0.451	0.467	0.477	0.486

**Table 3:** RMSE of the Costas Loop with Different Filter Coefficients.

$\alpha_{\omega }$	None	0.01	0.05	0.1	0.2	0.4	0.8	1.0
Costas loop	2.056	0.998	0.757	0.666	0.709	0.991	1.659	2.056

## Data Availability

The materials supporting the conclusions of this article, including the simulation code, main simulation parameter settings, generated datasets, and processed result data used for plotting and statistical analysis, are available from the corresponding author upon reasonable request. Since the algorithmic procedure, signal model, and simulation settings have been described in detail in the manuscript, readers are also encouraged to build their own simulation platform for reproduction and validation based on the information provided in this paper.
